# Diminished Estrogen Induced Mitochondrial Protection and Immunosuppressive Microenvironment in Gastric Cancer with Depression

**DOI:** 10.3390/cancers17172789

**Published:** 2025-08-26

**Authors:** Yixin Liu, Sheng Tian, Yujia Tan, Picheng Yan, Pan Liu, Huiying Zhu, Sachiyo Nomura, Tianhe Huang, Yongchang Wei

**Affiliations:** 1 Department of Radiation and Medical Oncology, Zhongnan Hospital of Wuhan University, Wuhan University, Wuhan 430071, China; 2 Hubei Key Laboratory of Tumor Biological Behaviors, Zhongnan Hospital of Wuhan University, Wuhan University, Wuhan 430071, China; 3 Hubei Cancer Clinical Study Center, Zhongnan Hospital of Wuhan University, Wuhan University, Wuhan 430071, China; 4 Second Clinical Hospital of Wuhan University, Zhongnan Hospital of Wuhan University, Wuhan University, Wuhan 430071, China; 5 Department of Gastrointestinal Surgery, Graduate School of Medicine, The University of Tokyo, Tokyo 113-8655, Japan; sachiyo.nomura1012@gmail.com

**Keywords:** gastric cancer, depression, estrogen, NOTCH3

## Abstract

Background: It is established that depression significantly contributes to tumor development, yet its molecular link to gastric cancer progression remains unclear. Methods: In this study, we examined depression-related gene expression profiles in relation to clinical prognosis and identified estradiol and the NOTCH3 gene as critical factors involved in gastric cancer progression in the context of depression. Using a chronic unpredictable stress-induced tumor-bearing mouse model, we validated the impact of depression on tumor development. Additionally, the underlying molecular mechanisms were explored through a range of biological techniques, including Western blotting, immunofluorescence, flow cytometry and immunohistochemistry. Results: Depression significantly accelerated gastric cancer growth in our mouse model, characterized by decreased estradiol levels and increased NOTCH3 expression. Importantly, exogenous estradiol supplementation effectively counteracted depression-induced tumor growth. Consistently, in vitro studies showed that estradiol treatment suppressed NOTCH3 expression in HGC-27 and YTN3 cell lines. Furthermore, NOTCH3 was shown to modulate intracellular reactive oxygen species levels by regulating SOD2 activity, thereby influencing cell proliferation. Conclusions: This work identified the estrogen/NOTCH3 signaling as a key link between depression and gastric cancer development, offering promising therapeutic strategies to improve outcomes for patients suffering from psychological disorders.

## Simple Summary

Depression is known to worsen outcomes in many cancer types, but its biological influence on gastric cancer has been unclear. This study reveals how depression promotes gastric cancer growth by disrupting hormone signaling and activating specific cancer pathways. Utilizing multiple approaches, we demonstrated that depression accelerated tumor growth, correlating with reduced systemic estradiol levels and upregulated NOTCH3 expression. Mechanistically, NOTCH3 was found to shape an immunosuppressive microenvironment and sustain cell proliferation through modulating reactive oxygen species homeostasis via SOD2 modulation. This work identifies the estrogen/NOTCH3 signaling as a key link between depression and gastric cancer, offering promising therapeutic strategies to improve outcomes for patients suffering from depression.

## 1. Introduction

Cancer remains a major obstacle to increasing life expectancy. According to GLOBOCAN 2022 statistics, gastric cancer (GC) ranks fifth worldwide in both incidence and mortality among all malignant tumors [1]. Patients with cancer frequently suffer from mental disorders such as fatigue, anxiety, fear and depression. Chronic psychological stress has been demonstrated to accelerate tumor progression and worsen clinical outcomes for patients with cancer [2,3,4]. Notably, approximately 40% of patients with GC reported depressive symptoms. However, the precise mechanisms linking it to poor prognosis remain elusive [5,6,7].

Sustained exploration in psychoneuroimmunology over the past decades have highlighted the pivotal role of the hypothalamic—pituitary—adrenal (HPA) axis and the sympathetic nervous system (SNS) in stress-mediated tumor development. These systems regulate various tumor-related biological processes, such as angiogenesis, neural remodeling and immune evasion [8,9]. Interestingly, sex hormones are also implicated in the stress response, implying their potential involvement in the intricate relationship between psychological stress and tumor biology [10,11]. Estrogen carries out diverse physiological functions ranging from reproductive system regulation to modulation of bone density, neuroplasticity and lipid metabolism. It is also involved in the development of certain types of tumors, particularly hormone-sensitive cancers like breast and endometrial cancer [12,13]. In light of these established foundations, it is essential to further explore their interplay.

NOTCH signaling is involved in the regulation of cell fate, organ development and tissue homeostasis in metazoans [14]. Among its subtypes, NOTCH3 has garnered significant attention for its dualistic role in cancer, functioning as an oncogene in most contexts and as a tumor suppressor in others [15,16,17,18,19]. Stress-induced neuroendocrine hormones always bind to specific receptors, triggering changes in the expression of cancer-related genes and activating intracellular signaling cascades. In this work, we identified NOTCH3 as a critical gene involved in stress-induced GC development. Depression was shown to decrease endogenous estradiol levels, resulting in the upregulation of NOTCH3, which facilitated GC progression by decreasing mitochondrial injury and shaping an immunosuppressive microenvironment. These data provide, to our knowledge, a novel pathway that explains psychological disorders and tumor progression.

## 2. Methods and Materials

### 2.1. Data Source

RNA sequencing data and relevant clinical information from The Cancer Genome Atlas (TCGA) were downloaded from the UCSC Xena browser (xenabrowser.net). Depression-related genes (DRGs) were obtained from the MalaCards database. The expression profiling of tumors in gastric adenocarcinoma patient-derived xenograft (PDX) mice model (GSE262056) was obtained from the Gene Expression Omnibus (GEO) database, and differentially expressed genes (DEGs) were obtained from the ‘Deseq2’ [20] package.

### 2.2. Analysis of Depression-Related Genes and Cluster Analysis

The union of genes associated with major depressive disorder, depressive disorder, melancholic depression, dysthymic disorder, bipolar disorder, atypical depressive disorder, mood disorder and anxiety in MalaCards (www.malacards.org, accessed on 15 January 2024) database was identified as depression-related genes (*n* = 351, Table S1). The ‘Deseq2’ package was used to calculate the fold change and Wald test values of depression-related gene expression in tumor and normal tissue for pan-cancer. Genes with a *p*-value < 0.05 were considered to be differentially expressed. The rank–rank hypergeometric overlap (RRHO) test [21] was utilized to assess the extent of overlap in depression-related gene signatures across different cancer types. DRGs that were differentially expressed in over 20 types of cancer were selected for cluster analysis (*n* = 20). Patients were stratified into two depression-related molecular subtypes using the ‘ConsensusClusterPlus’ [22] package. We conducted Kaplan–Meier (K-M) analysis and univariate Cox regression analysis to assess prognostic differences between the two clusters.

### 2.3. Cell Culture and Transfection

The human GC cell lines HGC-27 and AGS were obtained from the China Center for Type Culture Collection (Shanghai, China). The mouse YTN3 GC cell line was generously provided by Prof. Sachiyo Nomura from the University of Tokyo [23]. All cell lines were routinely tested and confirmed negative for mycoplasma contamination by DAPI staining. Cells were maintained in a humidified atmosphere containing 5% CO_2_ in either RPMI-1640 (HGC-27, AGS; Procell) or high glucose DMEM (YTN3; Procell) medium supplemented with 10% FBS (Gibco, Grand Island, NY, USA) and 1% penicillin/streptomycin (Biosharp, Hefei, China).

The shRNA plasmid targeting NOTCH3, packaged into lentivirus, was synthesized by Tsingke Biotechnology (Beijing, China). Cells were transduced with lentiviral particles and selected with puromycin (2 μg/mL) for seven days. Transient transfections were performed by using polyethylenimine (Beyotime, Shanghai, China) reagent according to the manufacturer’s protocol. A complete list of shRNA sequences can be found in Table S2.

### 2.4. Cell Proliferation Assay

HGC-27 and YTN3 cells were seeded into 96-well plates and incubated at 37 °C at a density of 1 × 10^3^ cells/well. Then, 24 h after estrogen treatment, 10 μL of CCK-8 reagent (Cat# MA0218, Meilunbio, Dalian, China) was added to each well and further incubated at 37 °C for 2 h. The absorbance at 450 nm was then measured using a microplate reader (SpectraMax M2, Molecular Devices, San Jose, CA, USA). Additionally, EdU assay was also performed to assess cell proliferation using a commercial kit (Cat# C0071, Beyotime, China). Cells were prepared as described previously [24], and fluorescence microscopy (Olympus, Tokyo, Japan) was used for detection.

### 2.5. Clone Formation Assay, Cellular ROS Assay and Mitochondrial Membrane Potential Assays

For clone formation assay, 500 cells/well were seeded in 6-well culture plates and incubated for 7–10 days after different treatment. When visible colonies were observed under an inverted microscope (Olympus, Japan), the cells were fixed with 4% paraformaldehyde and stained with 0.1% crystal violet solution for 15 min. Digital images of the colonies were captured, and the number of colonies was quantified using ImageJ software (NIH, Bethesda, MD, USA).

Cellular reactive oxygen species (ROS) levels were measured using a commercial kit. Briefly, cells were collected and incubated with the fluorescent probe 2,7-dichlorodihydrofuorescindiacetate (DCFH-DA, Cat# MA0219, Meilunbio, China) for 30 min at 37 °C in the dark. ROS levels were then detected using fluorescence microscopy or flow cytometry (Cytoflex, Beckman, Brea, CA, USA).

Mitochondrial membrane potential (MMP) was measured using the JC-1 probe (Beyotime, Cat# C2006, China). Cells were collected and stained with JC-1 dye for 20 min at 37 °C in the dark. According to the product instructions, JC-1 can aggregate in the matrix of mitochondria to form polymers with red fluorescence at high mitochondrial membrane potential, while existing as monomers with green fluorescence. The changes in MMP were measured by the percentages of JC-1 monomers.

### 2.6. Real-Time Quantitative Polymerase Chain Reaction (RT-qPCR) and Immunoblotting

Total RNA was extracted using TRIzol reagent (Agbio, Cat# AG21101, Changsha, China), and 1 ng of RNA was used for reverse transcription with the Hifair II 1st strand cDNA synthesis supermix (Cat# 11120ES60, Yeasen, Shanghai, China). Target gene expression levels were calculated using the delta Ct method and normalized to GAPDH. The oligonucleotide primer sequences are listed in Table S2.

For immunoblotting, total protein was obtained using RIPA buffer supplemented with protease inhibitor cocktail and subjected to SDS-PAGE as described previously [25]. Primary antibodies were incubated at 4 °C overnight, followed by a 1 h incubation with HRP-conjugated secondary antibodies at room temperature. Immunoreactivity was detected using chemiluminescence. The antibodies used in this study are listed in Table S3.

### 2.7. RNA Sequencing and Analysis

HGC-27 and AGS cells were cultured in Petri dishes and treated with β-Estradiol (E2, Solarbio, Cat# E8140, Beijing, China) for 24 h. Total RNA was extracted using TRIzol reagent, and RNA sequencing was performed by Tsingke Biotechnology (Beijing, China). After removing ribosomal RNA, PolyA-tailed RNA was fragmented into small pieces and reverse-transcribed to single-strand cDNA. The cDNA was ligated to adaptors, and sequencing was conducted on the DNBSEQ platform (BGI, Shenzhen, China). Differential gene expression analysis was performed using the edge package (v4.0).

### 2.8. Animal Procedures

Six-week-old female C57BL/6 mice were purchased from the Hubei BIONT Biological Technology (Wuhan, China). All animal experiments were approved by the Experimental Animal Welfare Ethics Committee of Zhongnan Hospital of Wuhan University (Approval No. ZN2023262). Mice were divided into the following four groups by a computer-generated random number sequence: shCtrl (NOTCH3 Ctrl), NOTCH3 shRNA#1 (NOTCH3 KD), shCtrl with CUS treatment (CUS+Ctrl) and NOTCH3 shRNA#1 with CUS treatment (CUS+KD). A chronic unpredictable stress (CUS) protocol was utilized to create the depression model [26]. Mice subjected to CUS treatment were randomly exposed to one of eight stressors (cage shaking for 5 min, restraint in a well-ventilated 50 mL polypropylene tube for 4 h, cage tilting to 45 degrees for 24 h, wet bedding for 24 h, social crowding for 24 h, food or water deprivation for 24 h, swimming in 4 °C ice water for 4 min and tail nipping for 5 min) at various times throughout the day, ensuring no stressor was repeated on consecutive days. Depression levels of mice were detected by elevated plus maze (EPM), open field test (OFT) and tail suspension test (TST). Behavioral data were collected and analyzed using Ethovision XT 17.5 software (Noldus, Leesburg, VA, USA).

YTN3 cells were suspended in 50% Matrigel and subcutaneously implanted into mice (8 × 10^6^ cells). Tumor volume was measured every three days and calculated with the formula ([width]^2^ × [length]/2). For the estrogen supplementation experiment, estradiol benzoate (EB, Solarbio, Cat# E8430, China) dissolved in sesame oil was administered for three weeks (0.5 μg/d, s.c.). The control animals were administered the same volume of sesame oil as the EB-treated group. Due to the distinct characteristics of the treatments, the personnel administering could not be blinded to group assignment. However, blinding was strictly implemented for all outcome assessments. Researchers performing behavioral tests, histological scoring and statistical analysis were kept unaware of group identities until all data were collected and analyzed.

### 2.9. Flow Cytometric Analysis and Immunohistochemistry

Single-cell suspensions were collected and labeled with specific antibodies. Death cells were identified using BD Horizon™ Fixable Viability Stain 510 (FVS510). The data were acquired on the Cytek Aurora/NL spectral flow cytometer (Cytek Biosciences, Fremont, CA, USA) and analyzed using FlowJo^TM^ v10.8.1 software (BD Biosciences, Franklin Lakes, NJ, USA). The reagents used in flow cytometry are listed in Table S2. Immunohistochemistry (IHC) was performed to assess the expression of various proteins in mice tumor tissues, following protocols described in previous studies [27]. Images were captured and analyzed using a Leica microscope and Aperio ImageScope software (Leica Biosystems, Wetzlar, Germany, v12.3.4.5008). For quantitative assessment, five random high-power fields (HPFs, 400× magnification) per sample were selected to evaluate specific staining metric. The field selection protocol avoided overlapping areas and excluded tissue edges to prevent bias.

### 2.10. Enzyme-Linked Immunosorbent Assay (ELISA)

To address the potential impact of CUS on circulating E2 levels and to confirm the efficacy of exogenous E2 supplementation in mice, serum concentrations were measured using a competitive ELISA method according to the manufacturer’s instructions (Biology, Cat# YPJ1186, Wuhan, China). Briefly, all reagents were equilibrated to room temperature prior to use. A total of 50 µL of calibrators or samples was added to the designated wells, followed by 50 µL of streptavidin/horseradish peroxidase (HRP)-labeled enzyme conjugate. The microplate was gently shaken for 30 s to ensure thorough mixing, then covered and incubated at 37 °C for 1 h. After thorough washing, 50 µL of hydrogen peroxide and 50 µL of 3,3′,5,5′-tetramethylbenzidine (TMB) buffer were added and the plate was then incubated at 37 °C in the dark for 15 min. The reaction was stopped by adding 50 µL of stop solution, and absorbance was measured at 450 nm. Sample concentrations were calculated based on calibration curve.

### 2.11. Quantification and Statistical Analysis

All statistical analyses and visualizations were conducted using R version 4.3.1 (R Foundation for Statistical Computing, Vienna, Austria). The R packages ‘survival’ (CRAN.R-project.org/package=survival) and ‘survminer’ were employed for univariate Cox proportional hazard regression and Kaplan–Meier survival analyses. X-tile [28] software was used to determine the optimal cut-off for survival analyses. Statistical differences between Kaplan–Meier survival curves were assessed using the log-rank test. Additionally, the R package ‘clusterProfiler’ [29] facilitated Gene Ontology (GO) and Kyoto Encyclopedia of Genes and Genomes (KEGG) enrichment analyses. For RNA-seq data, differentially expressed genes (DEGs) were defined as genes with |log2 fold-change| > 1 and adjusted *p*-value (FDR) < 0.05, using the Benjamini–Hochberg method for multiple testing correction. Data comparisons between two groups utilized the Wilcoxon test. Student’s t-test was performed to compare the means between two groups. Multiple group comparison was conducted by one-way analysis of variance (ANOVA). Two-way ANOVA was used for testing the statistical difference in cell viability. Statistical analysis was performed using GraphPad Prism 8.0 software. A *p*-value less than 0.05 was considered statistically significant for single comparisons, while the FDR correction was applied to multiple comparisons, with a q-value < 0.05 set as the significance threshold.

## 3. Result

### 3.1. NOTCH3 Was Identified as a Strong Indicator for Overall Survival and the Immune-Suppressive Tumor Microenvironment of Gastric Cancer

**Figure 1:**
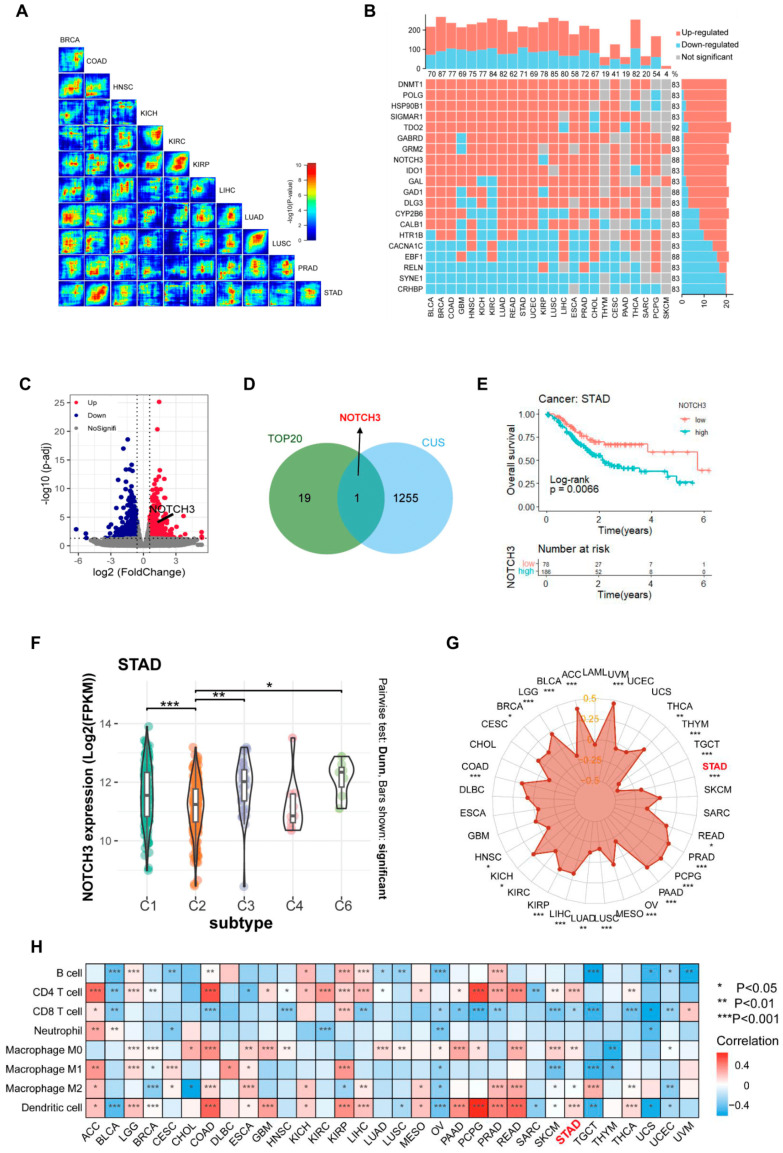
NOTCH3 was identified as a strong indicator for overall survival and the immune-suppressive tumor microenvironment of GC. (**A**) RRHO maps compare transcriptional profiles of depression-related genes across different tumor types, with the significance of overlap depicted in the color bar alongside the maps. (**B**) Summary statistics from differential expression analysis using DESeq2 of depression-related genes in tumor versus normal tissue across pan-cancer revealed the top twenty consistent DEGs. *p* < 0.05 for all up- and downregulated genes. (**C**) Volcano plot depicting DEGs in the CUS model, highlighting NOTCH3. (**D**) Venn diagrams displaying the overlap between depression-related genes from PDX model treated by CUS (blue) and top 20 consistent depression-related genes (green). (**E**) Overall survival Kaplan–Meier curves of patients with STAD divided by NOTCH3 (TCGA database). (**F**) Relationship between NOTCH3 expression and immune subtypes in STAD. (**G**) Radar charts illustrate the correlation between NOTCH3 expression and immune score (highlighting STAD). (**H**) The heat map illustrates the correlation between NOTCH3 expression and immune cell infiltration across pan-cancer (highlighting STAD). B cell, CD4 T cell and dendritic cell infiltration were calculated using TIMER, while all other infiltrates were assessed using CIBERSORT. * *p* < 0.05, ** *p* < 0.01, *** *p* < 0.001.

To clarify the molecular links between GC and depression, we retrieved 351 DRGs from the MalaCards human disease database. Subsequently, an unbiased RRHO analysis was applied to these genes across cancer types based on TCGA transcriptomic data. Figure 1A shows the consistency in expression of DEGs across different tumors as calculated by RRHO. This result indicates that there are always common changes in DRGs in the tumor RNA-Seq data of various cancer types. Among them, the digestive system cancers (COAD, STAD, LIHC) showed strong mutual overlap significance. In Figure 1B, a combined heatmap and bar graph representation illustrates the differential expression of DRGs across multiple tumor types. Overall, more than 70% of DRGs showed significant differential expression in all cancer types examined. Notably, the top 20 DRGs were differentially expressed in over 80% (20/24) of cancer types, with characteristics of consistent upregulation or downregulation. We also constructed a signature with the top 20 genes, and found that the signature was a prognostic factor for several cancer types, including GC (Figure S1). These findings imply a broad involvement of DRGs in pan-cancer tumorigenesis and progression.

Previously, we performed RNA-sequencing (RNA-Seq) on GC patient-derived tumor xenografts (PDXs), comparing tumor tissues from mice subjected to chronic unpredictable stress (CUS) with unstressed controls (GSE262056, Figure 1C). To further identify key DRGs in GC progression, we compared the top 20 depression related genes in cancer with our sequencing data from the PDXs, and NOTCH3 was the only overlapping gene (Figure 1D). Univariate Cox regression analysis demonstrated NOTCH3 as an indicator for poorer overall survival in patients with stomach adenocarcinoma (STAD), as well as multiple other malignancies (Figure 1E). A previous comprehensive immunogenomic analysis classified TCGA samples into six immune subtypes [30]. NOTCH3 was most highly expressed in the C6 (TGF-β dominant) subtype (Figure 1F), which is characterized by enhanced TGF-β-related signaling, abundant stromal components and the poorest prognosis. Moreover, NOTCH3 expression level was significantly negatively correlated with the abundance of tumor immune cells, calculated using the ESTIMATE algorithm (Figure 1G). This observation was further validated using the TIMER 2.0 database, where NOTCH3 positively correlated with the infiltration of several cell populations (CD4+ T cells, M0/M2 macrophages, dendritic cells) but negatively correlated with CD8+ T cells in STAD (Figure 1H). These data indicate that NOTCH3 is a strong indicator for survival and the immune-suppressive tumor microenvironment of GC.

### 3.2. Knockdown of NOTCH3 Suppressed GC Progression in Mice with Depression

**Figure 2:**
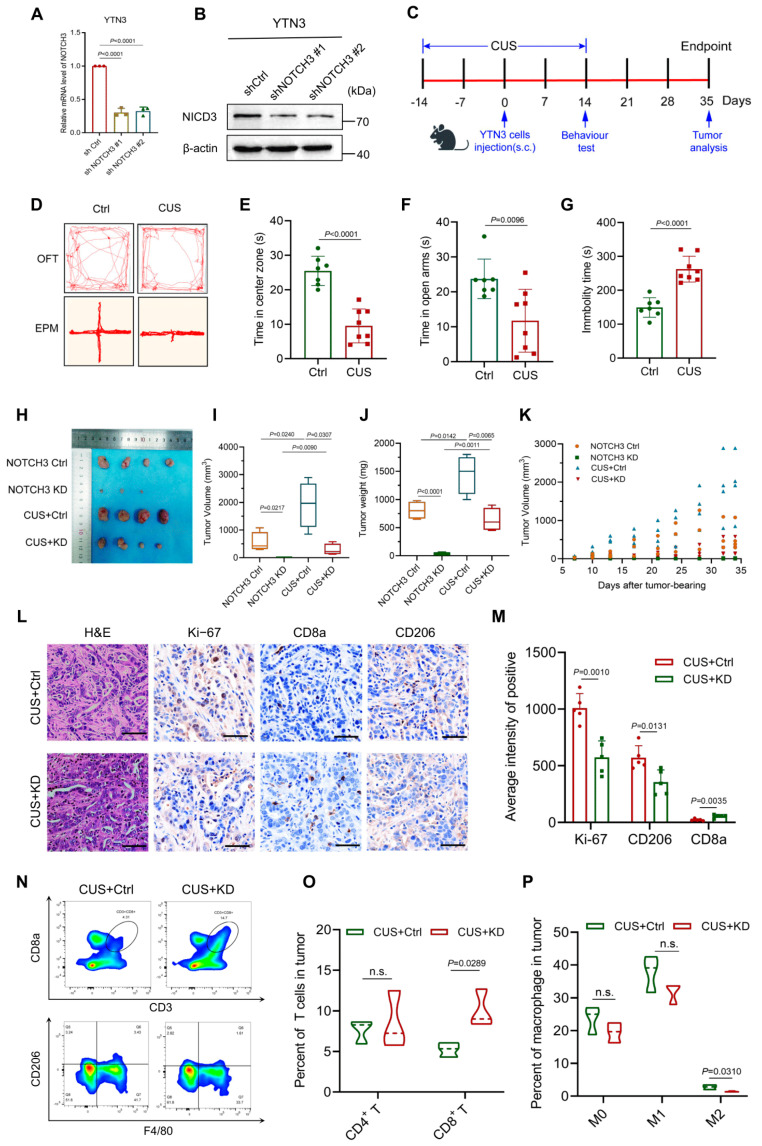
Knockdown of NOTCH3 suppressed GC progression in mice with depression. (**A**,**B**) Quantification of NOTCH3 knockdown efficiency in YTN3 cells with qRT-PCR and WB. (**C**) Flowchart of the animal experiments. (**D**) The representative movement trajectories of mice. (**E**–**G**) Depressive-like behaviors were assessed by the total time in center zone (**E**), total time in open arms (**F**) and total immobility duration (**G**). (**H**) Representative images of tumors in the 4 groups of NOTCH3 control, NOTCH3 KD, CUS and CUS + NOTCH3 KD (*n* = 4). (**I**) Tumor volume of the 4 groups (NOTCH3 control, NOTCH3 KD, CUS and CUS + NOTCH3 KD, *n* = 4). (**J**) Tumor weight of the 4 groups (NOTCH3 control, NOTCH3 KD, CUS and CUS + NOTCH3 KD, *n* = 4). (**K**) Tumor growth curve of the 4 groups (NOTCH3 control, NOTCH3 KD, CUS and CUS + NOTCH3 KD, *n* = 4). (**L**) Representative images of H&E staining and IHC staining of Ki-67, CD8 and CD206 (scale bar = 50 μm). (**M**) Comparison of Ki-67+ tumor cells, CD8+ cells and CD206+ cell in the tumor tissues from CUS treated tumor bearing mice with and without NOTCH3 knockdown (*n* = 5). (**N**–**P**) Analysis of CD8+ T cells, and CD206+ M2 in the tumor tissues from CUS-treated tumor-bearing mice with and without NOTCH3 knockdown with flow cytometry (*n* = 5).

To better understand the activities of NOTCH3 in GC with depression, we modified the NOTCH3 expression in the murine GC cell line YTN3 and the knockdown efficiency of NOTCH3 was successfully validated (Figure 2A,B). Before tumor cell injections, we induced the depressive behaviors of C57BL/6 mice by treating with CUS (Figure 2C). Behavioral assessments validated the successful establishment of the depression, as evidenced by less time spent in the center zone and the open arms, but increased immobility duration relative to the control group (Figure 2D–G). As demonstrated in Figure 2H–K, CUS treatment significantly enhanced tumor growth, as evidenced by increased tumor volume and weight. Notably, NOTCH3 knockdown effectively attenuated this effect, substantially suppressing tumor growth in both stressed and non-stressed cohorts. IHC analysis indicated that NOTCH3 knockdown suppressed tumor cell proliferation (fewer Ki-67+ cells) and promoted anti-tumor immunity (increased CD8+ T cell infiltration and diminished M2 macrophage accumulation) compared with controls (Figure 2L,M). These immunohistochemical findings were further corroborated by flow cytometry, which quantitatively confirmed the increased CD8+ T cell frequency and decreased M2 macrophage proportion in NOTCH3-deficient tumors (Figure 2N–P). Together, these data demonstrate that NOTCH3 depletion inhibits GC cell proliferation and reverses key features of tumor immunosuppression in the context of depression.

### 3.3. Activation of NOTCH3 Was Driven by the Diminished Estrogen in GC with Depression

**Figure 3:**
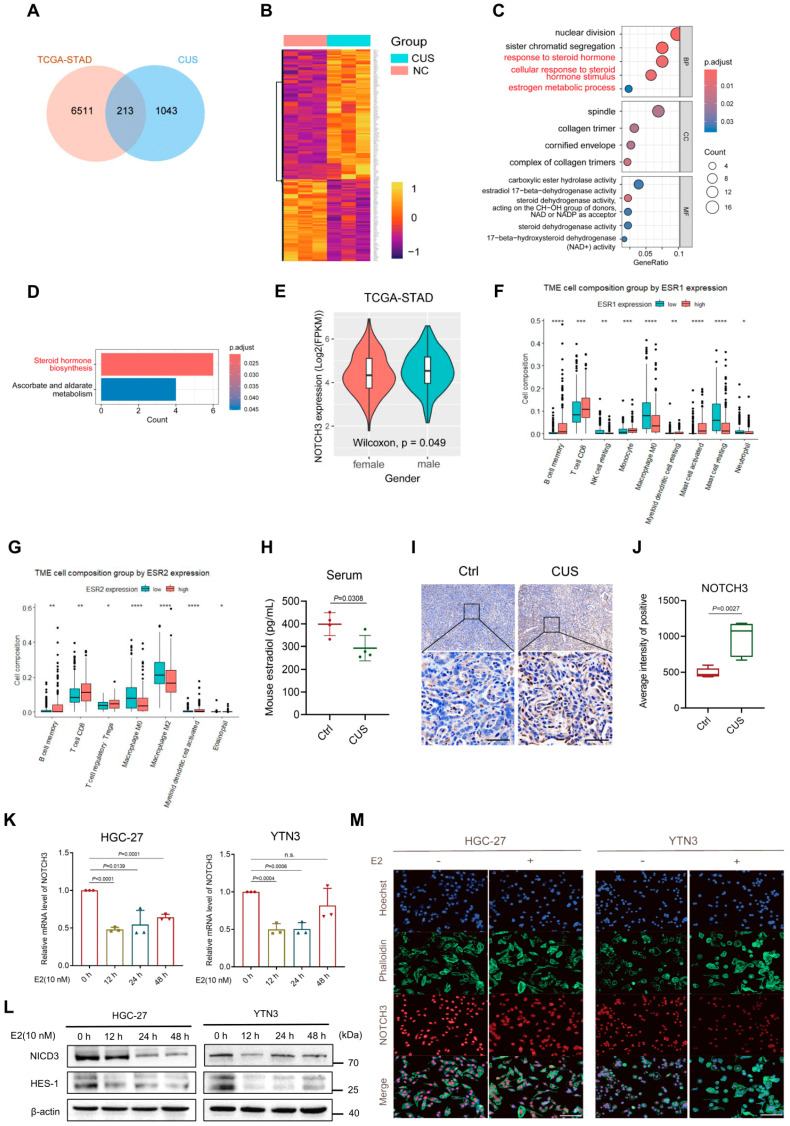
Activation of NOTCH3 was driven by diminished estrogen in GC with depression. (**A**) Venn diagrams displaying the overlap between DEGs in TCGA-STAD (orange) and depression-related genes from PDX model treated by CUS (blue). (**B**) Heatmap of the 213 overlapped DEGs in the tumor tissues from mice with and without CUS treatment. (**C**) GO enrichment analysis of the 213 overlapped DEGs. (**D**) KEGG pathway enrichment analysis of the 213 overlapped DEGs. (**E**) Comparison of NOTCH3 expression between female and male TCGA-STAD patients. (**F**,**G**) Tumor microenvironment cell composition grouped by ESR1 and ESR2 expression in TCGA-STAD (TIMER 2.0 database). (**H**) Serum estradiol levels in mice with and without CUS treatment *(n* = 4). (**I**,**J**) Histological analysis and NOTCH3 expression in tumor tissues from mice with and without CUS treatment (scale bar = 50 μm, *n* = 4). (**K**–**M**) Investigation of the effects of estradiol (E2) on NOTCH3 mRNA and protein levels in GC cell lines with RT-qPCR (*n* = 3), Western blotting assays and IF staining (10 nM for 24 h, scale bar = 50 μm). * *p* < 0.05, ** *p* < 0.01, *** *p* < 0.001, **** *p* < 0.0001.

To elucidate the mechanisms underlying NOTCH3 activation in depression-associated GC progression, we performed comparative transcriptomics between TCGA-STAD database and CUS-treated PDX cohorts, identifying that 213 overlapping DEGs were identified (Figure 3A,B). Functional enrichment analysis of these DEGs revealed significant enrichment in steroid hormone-related signaling pathways through both Gene Ontology (GO) and Kyoto Encyclopedia of Genes and Genomes (KEGG) analyses (Figure 3C,D). Interestingly, female patients with GC exhibited lower levels of NOTCH3 expression in the TCGA database (Figure 3E, *p* = 0.049), implying potential estrogen-mediated regulation of NOTCH3 signaling. Furthermore, TIMER 2.0 database analysis also revealed significant correlations between estrogen receptors and immune cell infiltration. As shown in Figure 3F,G, both estrogen receptor 1 (ESR1) and ESR2 showed positive associations with CD8+ T cell abundance, while exhibiting negative correlations with M0 macrophages. Only ESR2 displayed an inverse relationship with M2 macrophage infiltration. To mechanistically validate these correlations, we established a depression-associated GC mouse model, and ELISA analysis revealed significantly reduced serum estrogen levels in depressive mice (Figure 3H, *p* = 0.0308). IHC staining showed elevated NOTCH3 protein expression in CUS-exposed tumor tissues (Figure 3I,J, *p* = 0.0027). Consistently, treatment with estradiol (E2) significantly inhibited NOTCH3/HES1 signaling in HGC-27 and YTN3 cells (Figure 3K–M). These findings collectively establish that diminished estrogen can drive NOTCH3 pathway activation and foster an immunosuppressive microenvironment in GC with depression.

### 3.4. Estrogen Suppressed GC Growth and Induced Immunosuppressive Immune Cell Infiltration in the Depressive Mice

**Figure 4:**
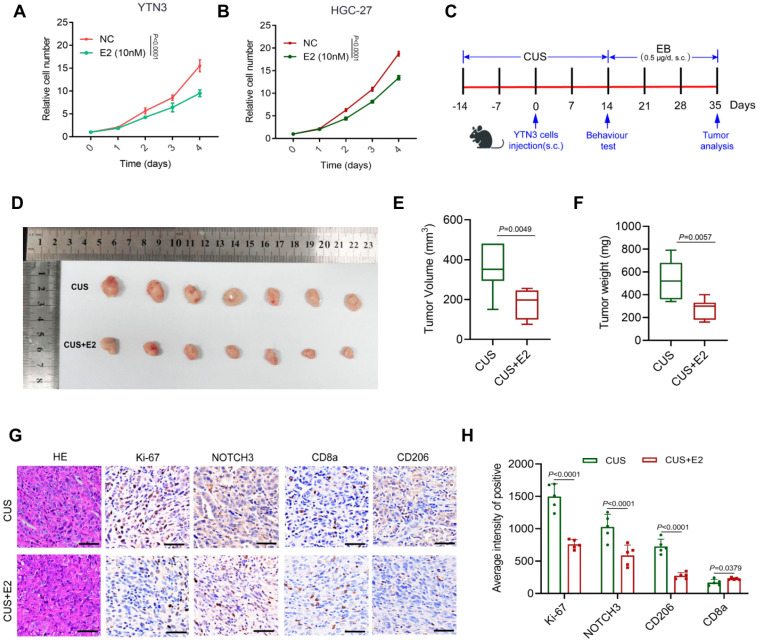
Estrogen suppressed GC growth and induced immunosuppressive immune cell infiltration in the depressive mice. (**A**,**B**) Proliferation curve of GC cell lines treated with E2. (**C**) Flowchart of the mouse experiments. (**D**) Representative images of tumor masses excised from CUS-treated mice with and without E2 supplement (*n* = 7). (**E**) Tumor volume of the 2 groups (CUS and CUS+E2, *n* = *7*). (**F**) Tumor weight of the 2 groups (CUS and CUS+E2, *n* = 7). (**G**) Representative images of H&E staining and IHC staining of Ki–67, NOTCH3, CD8a and CD206 (scale bar = 50 μm). (**H**) Comparison of Ki–67+ tumor cells, NOTCH3+ cells, CD8a+ cells and CD206+ cells in the tumor tissues of the 2 groups (CUS and CUS+E2, *n* = 5).

Given the previous observation of reduced E2 levels in the mouse model, we hypothesized that restoring estrogen signaling would attenuate depression-mediated tumor progression by targeting NOTCH3 driven pathways. To systematically evaluate E2’s therapeutic potential, we initiated our investigation with cell viability assays. E2 treatment significantly suppressed proliferation in HGC-27 and AGS (Figure 4A,B, all *p* < 0.0001), supporting its direct anti-tumor effects. We next extended these findings to the mouse in vivo model. As shown in Figure 4C–F, exogenous E2 supplementation effectively elevated circulating E2 levels (Figure S2A, *p* = 0.0006) and inhibited tumor volume (*p* = 0.0049) and weight (*p* = 0.0057). With IHC staining, we confirmed that E2 treatment decreased the Ki-67 positive tumor cells, reduced NOTCH3 expression, lessened M2 infiltration and increased CD8+ T cell infiltration (Figure 4G–H). Collectively, these results demonstrate that E2 supplementation not only directly inhibits GC growth but also reverses the immunosuppressive tumor microenvironment in the context of depression.

### 3.5. NOTCH3 Promoted Progression of GC Through Regulating SOD2 Activity

**Figure 5:**
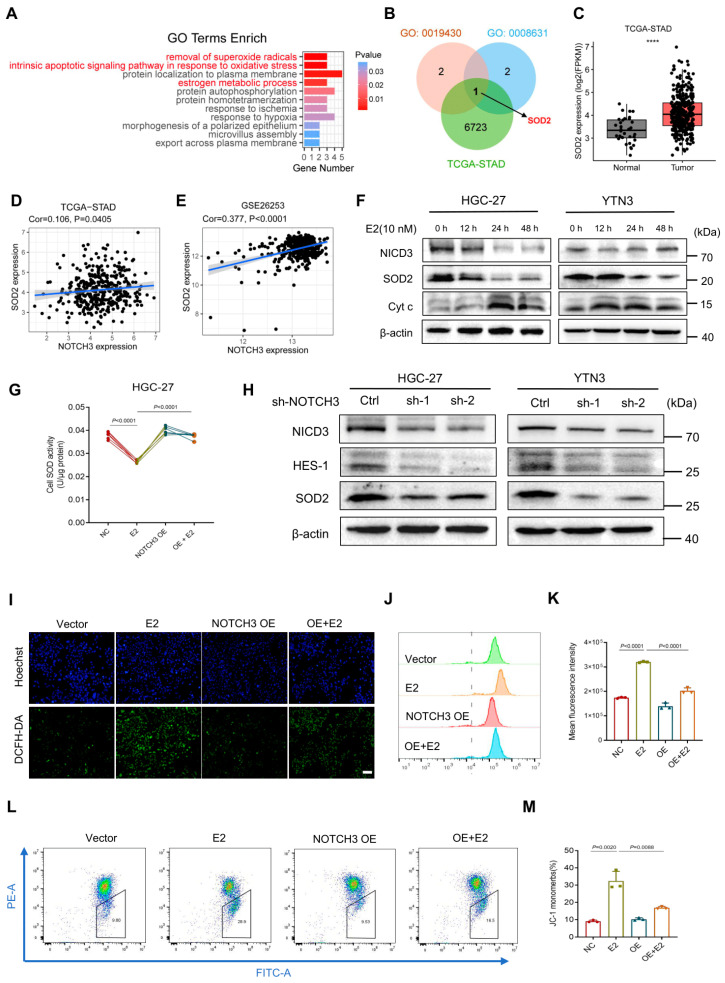
NOTCH3 promoted progression of GC through regulating SOD2 activity. (**A**) GO enrichment analysis of DEGs of E2-treated GC cells. (**B**) Venn diagrams displaying the overlap genes between TCGA-STAD (green), removal of superoxide radicals signaling (GO: 0019430, pink) and intrinsic apoptotic signaling pathway in response to oxidative stress (GO: 0008631, blue), highlighting SOD2. (**C**) Expression of NOTCH3 in tumor tissue (TCGA-STAD). (**D**,**E**) Analysis of correlation between NOTCH3 and SOD2 in TCGA-STAD and GSE26253 databases. (**F**) Detecting the effects of E2 on NOTCH3, SOD2 and cytochrome complex in GC cell lines with WB. (**G**) Detecting the effects of E2 and NOTCH3 OE on SOD activity in GC cells with SOD activity kit (*n* = 6). (**H**) Detecting the effects of NOTCH3 KD on SOD2 in GC cell lines with WB. (**I**) Representative images of DCFH-DA staining in HGC-27 cells (scale bar = 50 μm). (**J**,**K**) Comparison of ROS intensity in the HGC-27 cells of the 4 groups with flow cytometry (NC, E2, NOTCH3 OE and E2 + NOTCH3 OE, *n* = 3). (**L**,**M**) Mitochondrial membrane potential was measured using the JC-1 probe by flow cytometry (*n* = 3). **** *p* < 0.0001.

To elucidate the mechanisms by which E2 and NOTCH3 influence GC progression, we treated HGC-27 and AGS cells with E2 (10 nM, 24 h) and subjected them to RNA-seq analysis. GO enrichment analysis of DEGs indicated significant enrichment in pathways associated with superoxide removement and oxidative stress response (Figure 5A). Among these DEGs, SOD2 was notably enriched in the superoxide clearance pathway and found to be upregulated in patients with TCGA-STAD (Figure 5B). Malignant cells often exhibit high dependence on SOD, and Plunkett et al. found that estrogen suppressed tumor growth by inhibiting SOD activities [31]. This finding implicates that SOD may act as a downstream effector of E2-NOTCH3 signaling. Through the analysis of the TCGA-STAD and GEO26253 datasets, we found that SOD2 was markedly elevated in GC tissues (Figure 5C, *p* < 0.0001), with a positive correlation between NOTCH3 and SOD2 expression levels (Figure 5D,E). Furthermore, E2 treatment resulted in a decrease in SOD2 protein levels and an increase in the cytochrome complex (Figure 5F). Notably, the effect of E2 on SOD2 could be partially neutralized by NOTCH3 overexpression (Figure 5G). The overexpression efficiency of NOTCH3 in HGC-27 cells was successfully validated (Figure S2A,B). Conversely, the knockdown of NOTCH3 led to a suppression of SOD2 levels (Figure 5H), indicating that SOD2 is a direct target of NOTCH3 regulation. Besides the cytochrome complex, we also assessed the ROS levels and mitochondrial membrane permeability. Through immunofluorescence staining and flow cytometry, we confirmed that E2 treatment elevated ROS levels, while this effect could be dissipated by the activation of NOTCH3 (Figure 5I–K). Similarly, E2 induced mitochondrial membrane injuries, which could also be stabilized by NOTCH3 overexpression (Figure 5L,M). In summary, these data suggest that NOTCH3 facilitates the progression of GC by regulating SOD2 activity, thereby protecting the mitochondrial membrane from ROS-induced damage.

## 4. Discussion

In this study, we revealed that DRGs exhibited consistent dysregulation across multiple cancer types, suggesting their potential involvement in tumor progression and prognosis. Among these, NOTCH3 was identified as a key player in depression-induced GC growth, associated with poor prognosis and immune cell infiltration. Mechanistically, depression-induced estradiol reduction led to NOTCH3 upregulation, which prompted GC growth and altered immune cell composition, while exogenous estradiol supplementation alleviated this progression. In vitro experiments further revealed that estradiol supplementation inhibited GC cell proliferation by downregulating NOTCH3, which in turn reduced SOD2 activity, leading to ROS accumulation and mitochondrial injury. These findings underscore the critical role of NOTCH3 in depression-related GC progression and highlight estradiol as a potential therapeutic approach.

NOTCH3 is a single-pass transmembrane receptor composed of several distinct domains, including a large extracellular domain (NECD) composed of up to 34 EGF-like repeats, a transmembrane domain (TMD) and an intracellular domain (NICD). Upon binding of the NECD to its corresponding ligands (such as Jagged or Delta-like), two proteolytic cleavages release the NICD, which translocates to the nucleus to regulate gene expression [32,33]. In GC, studies have shown that NOTCH3 is highly expressed in tumor tissues and is associated with survival and immune infiltration [34,35], but there is limited research on the mechanisms by which NOTCH3 influences the biological behavior of GC. Previous studies [36,37] have also reported the relationship between NOTCH3 and ROS. The balance between oxidative and antioxidant systems is essential for rapid proliferation of tumor cells, while excessive ROS accumulation leads to DNA damage, cell death (apoptosis or ferroptosis) and irreversible cellular senescence [38]. In our study, NOTCH3 was identified as a key factor in depression-induced GC progression by regulating SOD2 activity and influencing cellular oxidative stress levels.

Cancer comorbidity with psychological disorders is common, with links to numerous adverse health outcomes [39]. Here, the consistent differential expression of DRGs across most tumor types highlights a complex interplay between mental health and cancer biology. In fact, depression can function both as a causative factor and a consequence of cancer. Tumor-associated depression (TAD) is characterized by complex interactions between the tumor microenvironment and host neuroimmune networks. Its underlying mechanisms mainly involve the activation of inflammatory responses during tumor development and treatment, which disrupt normal neurotransmission and impair neurogenesis [39,40]. More importantly, depression could act as a chronic stressor to accelerate tumor progression. With advancement in cancer neuroscience, the roles of the HPA axis and SNS dysfunction in chronic stress-mediated tumor progression have been thoroughly discussed [41,42]. For instance, stress-triggered glucocorticoid surges and Tsc22d3 upregulation undermines anticancer immunity by blunting dendritic cell type I interferon responses and suppressing IFN-γ^+^ T cell activation [43]. Adrenergic signaling also plays a critical role in this process [8,9,44]. In GC, β2-adrenergic activation promoted autophagic flux through the AMPK-ULK1 pathway to enhance cancer cell proliferation [45]. Moreover, depression accelerates GC invasion and metastasis by inducing a neuroendocrine phenotype via the catecholamine–ADRB2–MACC1 axis [46]. Here, we shift our focus to explore the potential role of estrogen instead of revisiting the well-established hormones such as catecholamines and glucocorticoids. Although not yet fully elucidated, the link between estrogen to GC has been increasingly recognized. Gender differences in incidence, coupled with the higher prevalence observed in postmenopausal women and those undergoing anti-estrogen therapy, suggest a protective role of estrogen against GC [47,48,49,50]. Additionally, strong evidence points to an association between depression and estrogen [51,52]. Estradiol supports positive mood by influencing the production and activity of neurotransmitters such as serotonin, dopamine and norepinephrine. Numerous clinical studies have reported that fluctuating estrogen levels increase the risk of depression in women, with patients with depression often exhibiting decreased estrogen levels [53,54,55].

The connection between estrogen and depression is relatively well-documented. Changes in estrogen levels during key life stages in women–such as puberty, the menstrual cycle, pregnancy, postpartum and menopause–can significantly influence the risk of developing depressive symptoms [51]. Additionally, female depressive patients exhibited lower estradiol level compared with healthy individuals [55,56]. A similar result was also found in our experiment, where estradiol levels were significantly reduced in tumor-bearing mice following CUS treatment. We next investigated the relationship between estradiol and NOTCH3, and found estradiol treatment could suppress the expression of NOTCH3 in HGC-27 and YTN3 cells. Moreover, exogenous estradiol supplementation inhibited tumor growth and markedly reduced NOTCH3 expression in tumor tissues. These findings suggest that estradiol may partly mediate depression-induced GC growth by regulating NOTCH3 expression.

Our work offers valuable insights into the role of E2 and NOTCH3 in depression-induced GC development; however, several limitations must be acknowledged. As a preclinical investigation, our findings should be validated with further clinical samples. Additionally, although E2 treatment attenuated GC progression in depressed mice, the lack of a non-depressed cohort leaves it unclear whether E2 exerts comparable anti-tumor effects in the absence of depression. Therefore, future studies should systematically compare tumor progression in both depressed and non-depressed mice models with and without E2 intervention to determine whether its therapeutic efficacy is specific to depressive condition. Moreover, the applicability of our research findings is limited to the gender-specific nature of the study subjects. It is also important to note that an opposite conclusion may be drawn in certain hormone-related tumors like breast cancer [11].

Hormonal cyclicity is another critical factor to be considered in this context. Physiological fluctuations in estrogen levels, such as those occurring during the estrous cycle, pregnancy or menopause, may significantly influence both depressive behaviors and GC progression, thereby introducing confounding variables not addressed under controlled experimental conditions. In our study, although the administrator of exogenous E2 at a fixed dose ensured methodological consistent, it may not adequately reflect complex natural hormonal variations. Consequently, our findings might represent only a narrow window of E2’s anti-tumor effects within a specific hormonal context. Future studies should incorporate longitudinal monitoring of endogenous estrogen levels and assessments across different hormonal stages to better elucidate how fluctuating E2 concentrations modulate disease progression.

## 5. Conclusions

In conclusion, we highlighted the critical role of NOTCH3 in linking depression and gastric cancer progression. Depression-induced estrogen reduction leads to the activation of NOTCH3, which promotes GC growth through shaping an immunosuppressive microenvironment and reducing mitochondrial injury.

## Data Availability

The datasets used and analyzed in this paper are available from the corresponding author upon reasonable request.

## Supplementary Materials

The following supporting information can be downloaded at: https://www.mdpi.com/article/10.3390/cancers17172789/s1, Figure S1: Relationship between depression-associated molecular subtypes and OS in pan-cancer. (A) Forest plot displayed relationship between subtypes and OS via univariate Cox analysis. All hazard ratios were calculated by comparing higher-risk subtypes to lower-risk ones. (B) The impact of subtypes on OS in the specified cancer types was evaluated using Kaplan–Meier survival curves; Figure S2: (A) Serum estradiol levels in mice with and without EB treatment. (B,C) Quantification of NOTCH3 overexpression efficiency in HGC-27 cells with qRT-PCR and WB; Figure S3: Original blots images; Table S1: Identified depression related genes in MalaCards; Table S2: shRNA and primers sequences; Table S3: Key resources table.

## Abbreviations

CCK-8	Cell counting kit-8
CUS	Chronic unpredictable stress
ELISA	Enzyme-linked immunosorbent assay
EPM	Elevated plus maze
GC	Gastric cancer
HPA	Hypothalamic—pituitary—adrenal axis
IHC	Immunohistochemistry
NICD	Notch intracellular domain
OFT	Open field test
OS	Overall survival
PDX	Patient-derived xenografts
RT-qPCR	Real-time quantitative polymerase chain reaction
shRNA	Short hairpin RNA
SNS	Sympathetic nervous system
STAD	Stomach adenocarcinoma
TST	Tail suspension test
