# Supplementary material for: Diminished Estrogen Induced Mitochondrial Protection and Immunosuppressive Microenvironment in Gastric Cancer with Depression
Source: Cancers (Basel). 2025 Aug 26;17(17):2789. doi: 10.3390/cancers17172789 (PMC12427221; doi:10.3390/cancers17172789)
Supplement: Supplementary file 1 [file cancers-17-02789-s001.zip › Figure S3.pdf]

Original Blots Image

Figure 2B

Left to right: YTN3 cells; shCtrl shNOTCH3#1 shNOTCH3#2

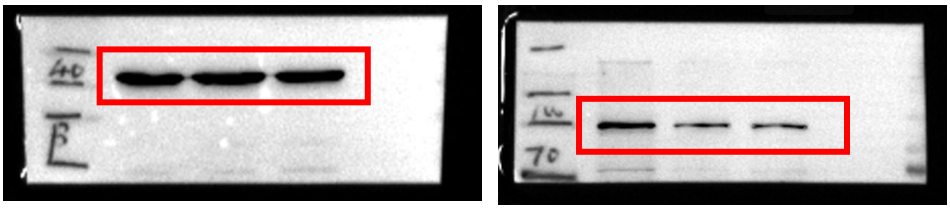

β-actin NICD3

Figure 3L

Left to right: HGC-27 cells; E2-0h E2-12h E2-24h E2-48h

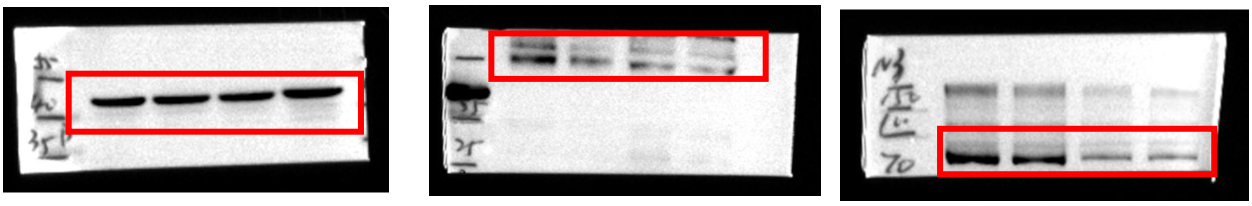

β-actin HES-1 NICD3

Left to right: YTN3 cells; E2-0h E2-12h E2-24h E2-48h

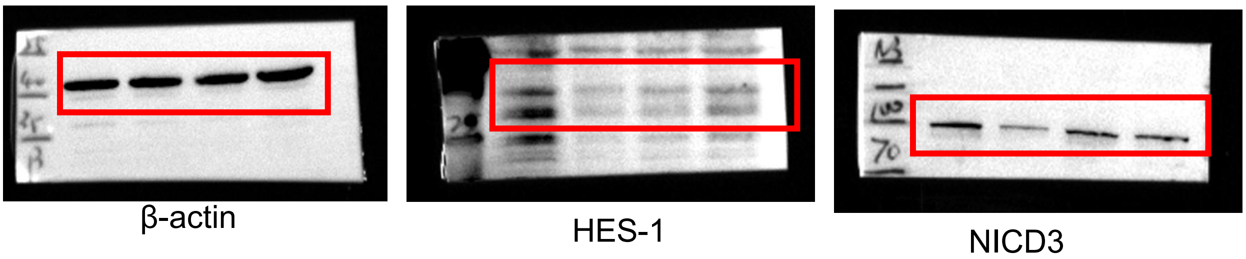

β-actin HES-1 NICD3

Figure 5F

Left to right: HGC-27 cells; E2-0h E2-12h E2-24h E2-48h

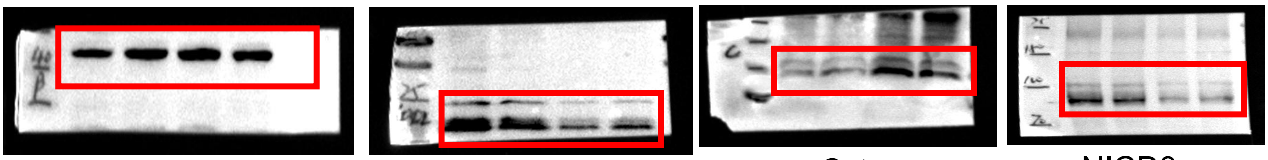

β-actin SOD2 Cyt c NICD3

Left to right: YTN3 cells; E2-0h E2-12h E2-24h E2-48h

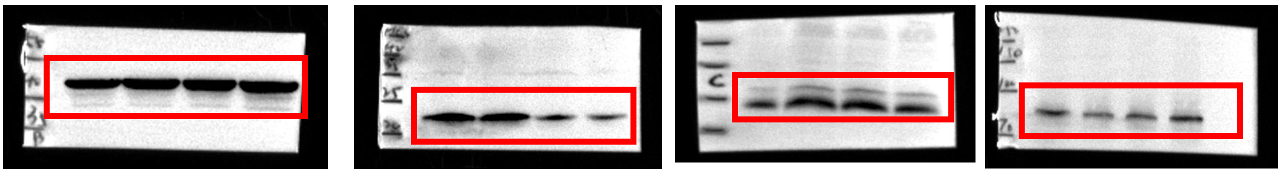

β-actin SOD2 Cyt c NICD3

Figure 5H

Left to right: HGC-27 cells; shCtrl shNOTCH3#1 shNOTCH3#2

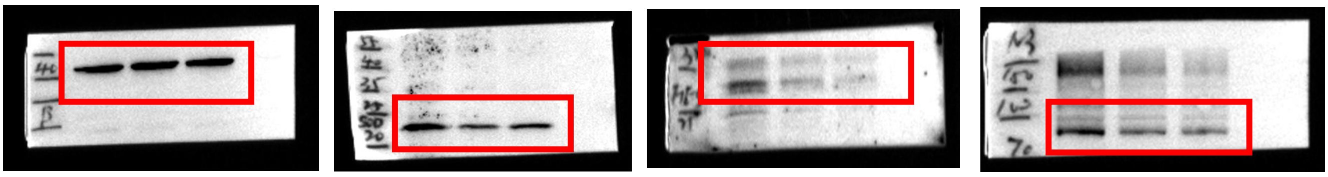

β-actin SOD2 HES-1 NICD3

Left to right: YTN3 cells; shCtrl shNOTCH3#1 shNOTCH3#2

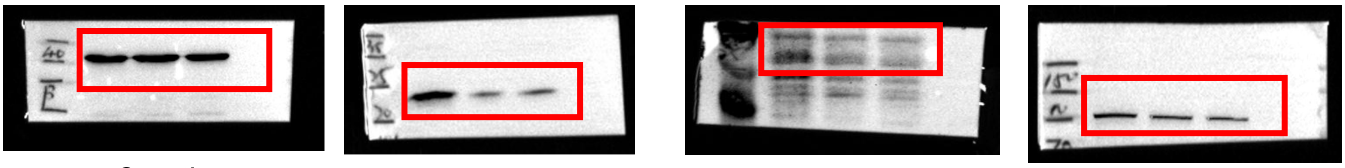

β-actin SOD2 HES-1 NICD3

Figure S2

Left to right: HGC-27 cells; Vector NOTCH3-OE Vector NOTCH3-OE

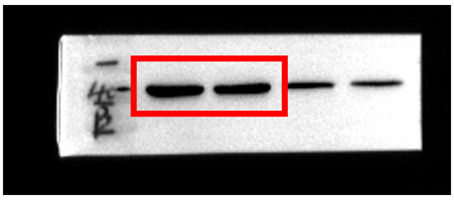

β-actin

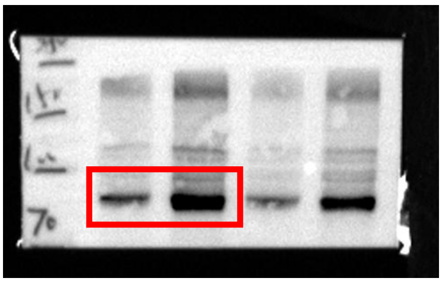

NICD3
