# Supplementary material for: Diminished Estrogen Induced Mitochondrial Protection and Immunosuppressive Microenvironment in Gastric Cancer with Depression
Source: Cancers (Basel). 2025 Aug 26;17(17):2789. doi: 10.3390/cancers17172789 (PMC12427221; doi:10.3390/cancers17172789)
Supplement: Supplementary file 1 [file cancers-17-02789-s001.zip › Table S2 and S3.pdf]

Table S2. shRNA and primers sequences

Mouse NOTCH3 shRNA#1	CCGGGCGTTCAGTGCCTACACAATGCTCGAGC ATTGTGTAGGCACTGAACGCTTTTTT
Mouse NOTCH3 shRNA#2	CCGGGGACAAGATGCACTGGGAATGCTCGAGC ATTCCCAGTGCATCTTGTCCTTTTTT
Human NOTCH3 shRNA#1	CCGGGGCTTCACAGGAACCTATTGCCTCGAGGCAATA GGTTCCTG
Human NOTCH3 shRNA#2	CCGGGCAGATGACACATCAGCTAGCCTCGAGGCTAGC TGATGTGTCATCTGCTTTTTT
shCtrl	CCGGTTCTCCGAACGTGTCACGTACGTGACACG TTCGGAGAATTTTTT
Human NOTCH3	Forward: 5′-TACTGGTAGCCACTGTGAGCAG-3′
	Reverse: 5′-CAGTTATCACCATTGTAGCCAGG-3′
Mouse NOTCH3	Forward: 5′-GGTAGTCACTGTGAACACGAGG-3′
	Reverse: 5′-CAACTGTCACCAGCATAGCCAG-3′
Human GAPDH	Forward: 5′-GTCTCCTCTGACTTCAACAGCG-3′
	Reverse: 5′-ACCACCCTGTTGCTGTAGCCAA-3′
Mouse GAPDH	Forward: 5′-CATCACTGCCACCCAGAAGACTG-3′
	Reverse: 5′-ATGCCAGTGAGCTTCCCGTTCAG-3′

Table S3 Key resources table

Reagent	Source	Identifier
β-actin	Proteintech	66009-1-Ig
SOD2	Zenbio	306028
HES-1	Beyotime	AF2167
NOTCH3	Zenbio	161043
Cytochrome C	Zenbio	250109
Ki-67	Proteintech	27309-1-AP
CD8a	Servicebio	GB15068-50
CD206	Proteintech	18704-1-AP
Hoechst 33342	Beyotime	C1025
Phalloidin	Yeasen	40736ES75
CD45-PE	Biolegend	103106
CD11b-APC	Biolegend	101212
F4/80-APC-Cy7	Biolegend	123117
CD86-PE/Cy7	Biolegend	105005
CD206-BV605	Biolegend	141721
CD3-APC	Biolegend	100236
CD4-APC-Cy7	Biolegend	100525
CD8-PE-Cy7	Biolegend	100721
FVS 510	BD Bioscience	564406
