# Supplementary figures and images for: Diminished Estrogen Induced Mitochondrial Protection and Immunosuppressive Microenvironment in Gastric Cancer with Depression
Source: Cancers (Basel). 2025 Aug 26;17(17):2789. doi: 10.3390/cancers17172789 (PMC12427221; doi:10.3390/cancers17172789)

Figure S1

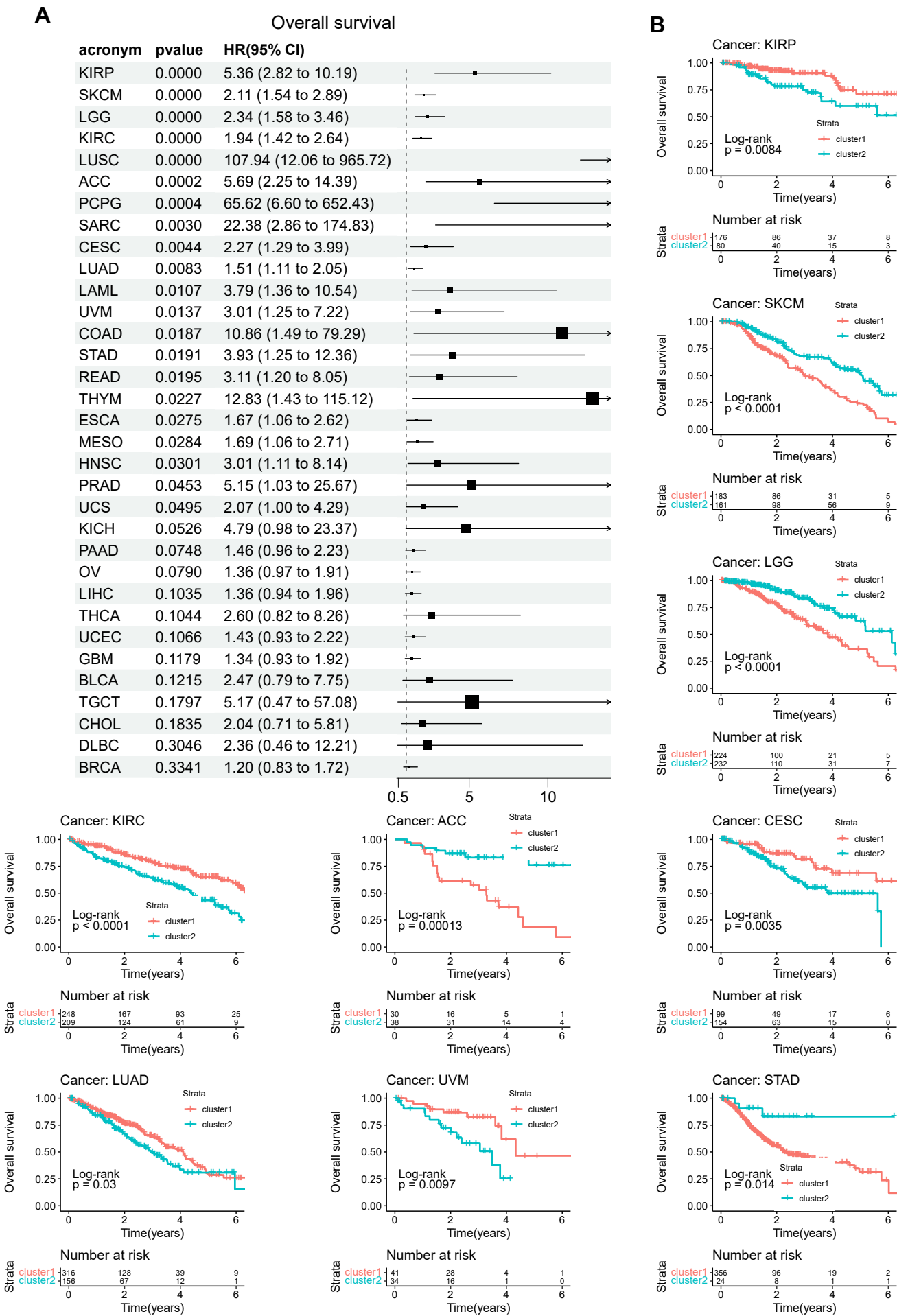

Figure S2

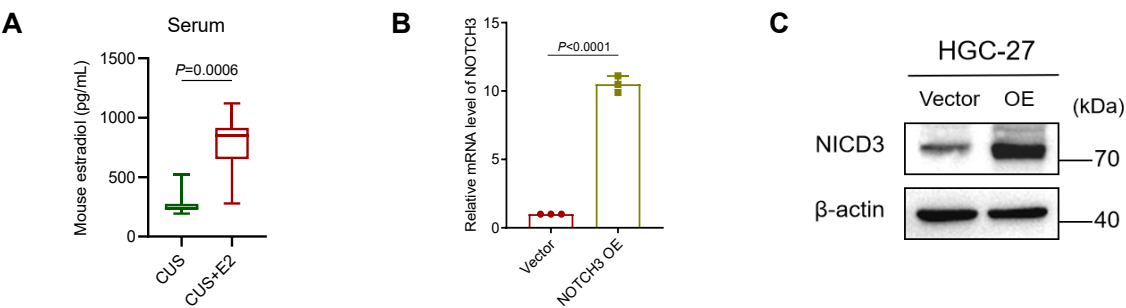

Supplement: Supplementary file 1 [file cancers-17-02789-s001.zip › Figure S1-S2.pdf]
